# Comparison of ciprofloxacin versus fosfomycin versus fosfomycin plus trimethoprim/sulfamethoxazole for preventing infections after transrectal prostate biopsy

**DOI:** 10.1007/s00345-024-05048-4

**Published:** 2024-05-28

**Authors:** Alberto Bovo, Maciej Kwiatkowski, Lukas Manka, Christian Wetterauer, Christoph Andreas Fux, Marco Cattaneo, Stephen F. Wyler, Lukas Prause

**Affiliations:** 1 Department of Urology, Cantonal Hospital Aarau, Tellstrasse 25, 5001 Aarau, Switzerland; 2 Medical Faculty, University Hospital Basel, Basel, Switzerland; 3 Department of Urology, Academic Hospital Braunschweig, Brunswick, Germany; 4 Department of Urology, Faculty of Health Sciences Brandenburg, Brandenburg Medical School Theodor Fontane, Potsdam, Germany; 5 Department of Urology, University Hospital Basel, Basel, Switzerland; 6 Department of Medicine, Faculty of Medicine and Dentistry, Danube Private University, 3500 Krems, Austria; 7 Department of Infectious Diseases and Hospital Hygiene, Cantonal Hospital Aarau, Aarau, Switzerland; 8 Department of Clinical Research, University of Basel, 4001 Basel, Switzerland

**Keywords:** Prostate biopsy, Prostate cancer, Periinterventional prophylaxis, Antibiotic prophylaxis

## Abstract

**Background:**

To evaluate antibiotic prophylaxis in transrectal prostate biopsies due to the recommendation of the European Medicines Agency (EMA): We describe our single center experience switching from ciprofloxacin to fosfomycin trometamol (FMT) alone and to an augmented prophylaxis combining fosfomycin and trimethoprim/sulfamethoxazole (TMP/SMX).

**Methods:**

Between 01/2019 and 12/2020 we compared three different regimes. The primary endpoint was the clinical diagnosis of an infection within 4 weeks after biopsy. We enrolled 822 men, 398 (48%) of whom received ciprofloxacin (group-C), 136 (16.5%) received FMT (group-F) and 288 (35%) received the combination of TMP/SMX and FMT (group-BF).

**Results:**

Baseline characteristics were similar between groups. In total 37/398 (5%) postinterventional infections were detected, of which 13/398 (3%) vs 18/136 (13.2%) vs 6/288 (2.1%) were detected in group-C, group-F and group-BF respectively. The relative risk of infectious complication was 1.3 (CI 0.7–2.6) for group-C vs. group-BF and 2.8 (CI 1.4–5.7) for group-F vs. group-BF respectively.

**Conclusion:**

The replacement of ciprofloxacin by fosfomycin alone resulted in a significant increase of postinterventional infections, while the combination of FMT and TMP/SMX had a comparable infection rate to FQ without apparent adverse events. Therefore, this combined regimen of FMT and TMP/SMX is recommended.

## Background

Transrectal ultrasound guided prostate biopsy (TRUS-PB) has been the standard approach for diagnosing prostate cancer, with around 1 million biopsies performed annually across Europe [1]. While this procedure is routinely performed safely in an outpatient setting, there is a small percentage of patients who experience complications such as rectal bleeding, urinary retention, urinary tract infections (UTI) and sometimes even severe cases of urosepsis [2, 3].

For this reason, several international guidelines have endorsed a transperineal prostate biopsy (TPB) approach as a less infectious route. This technique however, requires retraining and new costly equipment which makes it unavailable for the vast majority of patients in the foreseeable future. Therefore, the transrectal approach with periinterventional antibiotic prophylaxis will remain the most common method world-wide and optimizing the antibiotic prophylaxis regimen for this procedure remains important [4]. For example trends in the UK have shown that over the past decade, TRUS-PB has been far more commonly performed than TPB, at a ratio of almost 4:1 [5].

Since the 1980s, fluoroquinolones (FQ) have been widely utilized for periinterventional antibiotic prophylaxis, mainly because ciprofloxacin rapidly penetrates into prostate tissue and covers most of the relevant pathogens [6]. However, the increasing rates of fluoroquinolone resistance, coupled with mounting evidence of serious adverse effects such as polyneuropathy, myopathy or connective tissue damages [7], have led several authorities to raise concerns. As a result, in 2019 the European Medicines Agency (EMA) issued a general restriction for FQ use and withdrew the marketing authorization of certain rarely used FQ [8]. The same year, the Federal Institute for Drugs and Medical Devices in Germany ceased recommending FQs for antibiotic prophylaxis for interventions in the urogenital tract.

This directive led to the adoption of alternative antibiotics such as fosfomycin, cephalosporins, ertapenem, or to a targeted prophylaxis based on a rectal swab culture taken prior to the biopsy or according to local resistance data [9].

Fosfomycin, an antibiotic with a wide spectrum against Gram-negative and Gram-positive bacteria stands out in this new landscape due to its safety and effectiveness even against multidrug-resistant pathogens and chronic prostatitis [10]. Consequently, fosfomycin has been proposed as an alternative agent for antibiotic prophylaxis for prostate biopsies [1, 11–15].

In this paper we compare three different regimes of antibiotic prophylaxis for transrectal prostate biopsy: ciprofloxacin vs fosfomycin trometamol (FMT) vs FMT plus trimethoprim/sulfamethoxazole (TMP/SMX).

## Materials and methods

### Study design and settings, characteristics of participants

This is a retrospective study performed between 01/2019 and 12/2020. We analyzed complications in men who underwent transrectal prostate biopsies for suspected PCa or for active surveillance of known low risk PCa. Three different regimes of antibiotic prophylaxis were used in our center during that period. Indications for prostate biopsies were based on the European Association of Urology (EAU) Guidelines [16]. We performed totally 950 prostate biopsies in the above mentioned period. We excluded 69 patients who underwent transperineal biopsy and 59 patients who received a modified antibiotic prophylaxis, thus 822 patients were eligible for this analysis. The periinterventional workup included a complete urological history, clinical examination, multiparametric magnetic resonance imaging of the prostate (mpMRI) and PSA quantification.

### Procedure and postoperative evaluation

Biopsies were taken using a transrectal ultrasound system (HI VISION Preirus, Fujifilm Inc., Tokyo, Japan) supplied with an end-fire probe in a transrectal approach with the periprostatic nerve block (24 mL 1.0% mepivacaine). All patients underwent standard random 12-core prostate biopsy. In the presence of PIRADS Score ≥ 3 lesions in a mpMRI, an TRUS-MRI fusion biopsy (Artemis Fusion System, Eigen Health Inc., Grass Valley, CA, USA) was performed, with 2–4 additional biopsies per lesion. All biopsies were performed by a trained urologists, foremost (787/822, 95.7%) by MK.

The ciprofloxacin group (C) received a periinterventional antimicrobial prophylaxis consisting of oral ciprofloxacin 500 mg twice daily for 3 days (first dose administered in the evening of the day before the procedure).

The dose used for the fosfomycin group (F) was 3 g taken orally 2.5 h before the procedure and another one in the evening of the same day.

Due to an unexpectedly high infection rate in F group, we then switched to a combination of FMT and trimethoprim/sulfamethoxazole (TMP/SMX) (BF group).

For the BF group, fosfomycin was combined with 960 mg of oral TMP/SMX to be taken approx. 30 min. before the intervention and in the evening of the same day.

All patients were instructed to report any clinical signs of post TRUS-PB UTI and/or adverse events of the antibiotic prophylaxis.

Data was collected retrospectively by reviewing all outpatient and hospital medical charts including data of the general practitioners. All complications within 30 days after the intervention including macrohematuria, rectal bleeding, pain, dysuria, fever and chills, UTI, and urinary retention were recorded. Post TRUS-PB UTI was managed according to EAU guideline standards.

In line with EAU guidelines, we evaluated the following signs and symptom of a UTI:

- Dysuria, urgency, frequency, suprapubic tenderness and flank pain.
- Fever ≥ 38 °C accompanied by chills and malaise (without other clinical focus).
- Urosepsis as defined in the EAU guidelines: organ dysfunction represented by an increase in the Sequential [Sepsis-related] Organ Failure Assessment (SOFA) score of 2 points or more.

### Primary and secondary endpoints

We defined the primary endpoint as symptomatic UTI, characterized by signs and/or symptoms of an UTI. A positive urine culture, blood culture and urosepsis were considered secondary endpoints.

### Statistical analysis

Numerical variables were expressed in medians and interquartile ranges (IQRs), categorical variables as frequencies and proportions. Between-group differences were assessed using a Chi-squared test or Fisher’s exact test for categorical variables, whenever appropriate. A Kruskal–Wallis test was used for the comparison of continuous variables across different treatment groups (different antibiotics), and significantly associated variables were adjusted by the Bonferroni correction for multiple tests. For the comparison of numerical variables among patients with and without urinary tract infections (UTIs), a Mann–Whitney U test was applied. To assess the independent associations with UTI, variables potentially associated with UTI were subsequently used in a logistic regression model (uni- and multivariate) as independent variables, whereas the presence of UTI (no versus yes) was used as a dependent variable. The outcomes of the regression model were expressed as odds ratios (ORs) and their respective 95% confidence intervals (95%CIs). A p value of < 0.05 was considered statistically significant. Statistical analysis was carried out using the Statistical Package for the Social Sciences (IBM Corp. Released 2019. IBM SPSS Statistics for Windows, Version 26.0. Armonk, NY: IBM Corp) and R version 4.1.1.

## Results

### Patient characteristics

From January 2019 to December 2020, 822 men with transrectal biopsies were included; 398 (48.4%) received ciprofloxacin (group-C), 136 (16.5%) received FMT (group-F), and 288 (35.0%) received FMT plus TMP/SMX (group-BF) as periinterventional antibiotic prophylaxis.

Forty-eight (6%) patients received standard ultrasound-guided biopsies and 774 (94%) received additional targeted MR/ultrasound fusion biopsies. Table 1 shows the baseline characteristics of all study groups. Overall, 47 out of 822 (5.7%) patients developed complications after the procedure (5 had rectal bleeding, 5 had hematuria and/or acute urinary retention, 37 had an UTI).

**Table 1:** Baseline characteristics of patients. Values are medians and interquartile ranges (IQRs) or case frequencies and percentage (n(%))

Parameter	Overall (n = 822)	C-arm (n = 398)	F-arm (n = 136)	BF-arm (n = 288)
Age (years)	66.0 (61.0–70.5)	65.7 (61.6–70.5)	66.3 (61.0–70.4)	66.4 (60.6–70.6)
BMI (kg/m^2^)	25.9 (24.1–28.4)	25.9 (23.9–28.7)	26.6 (24.6–29.4)	25.7 (24.3–27.8)
PSA (µg/l)	5.7 (4.1–9.0)	6.0 (4.2–9.2)	5.6 (4.2–9.1)	5.2 (3.9–8.8)
Prostate TZV in TRUS	15.7 (9.5–25.2)	17.2 (10.3–29.6)	15.8 (9.1–24.9)	14.5 (8.8–22.5)
Prostate volume in TRUS	47.6 (34.5–66.4)	49.7 (35.7–69.4)	45.2 (32.1–64.4)	45.1 (33.9–63.2)
Prostate volume in MRI	47.0 (35.0–64.8)	48.2 (35.0–67.9)	47.0 (34.5–62.0)	47.0 (35.0–64.0)
History of prostate biopsy n (%)	279 (33.9%)	135 (33.9%)	46 (33.8%)	98 (34.0%)
Number of biopsy cores	20 (17–22)	20 (17–22)	21 (17–24)	19 (16–21)
Cancer diagnosis n (%)	455 (55.4%)	214 (53.8%)	79 (58.1%)	162 (56.3%)

*BMI* body mass index, *C-arm* ciprofloxacin arm, *F* Fosfomycin arm, *BF* bactrim plus fosfomycin arm, *PSA* prostate specific antigen, *TZV* transitional zone volume

### Clinical endpoints

The results of primary and secondary endpoints and their relative risks according to the different prophylaxis groups are demonstrated in Table 2. The overall incidence of post procedure/biopsy UTI was 37/822 (4.5%, 95% CI 3.3–6.1%), with significant differences between the C, F, and BF group (3.3% versus 13.2% versus 2.1%, respectively, p < 0.0001). Similarly, we observed significant differences concerning the incidence of fever (2.0% versus 11.0% versus 1.4%, respectively, p < 0.0001) and chills (2.3% versus 5.9% versus 0.7%, respectively, p = 0.004).

**Table 2:** Primary and secondary endpoints

Parameter	Overall (N = 822) N (%) [95% CI]	Study groups				RR [95% CI]		
		C-arm, N = 398	F-arm, N = 136	BF-arm, N = 288	p	C vs BF	F vs C	F vs BF
Primary endpoint								
UTI	37 (4.5%) [3.3–6.1%]	13 (3.3%)	18 (13.2%)	6 (2.1%)	** < 0.0001**	1.3 [0.7–2.6]	1.8 [1.2–2.8]	2.8 [1.4–5.7]
Fever	27 (3.3%) [2.3–4.7%]	8 (2.0%)	15 (11.0%)	4 (1.4%)	** < 0.0001**	1.3 [0.6–2.8]	2.2 [1.3–3.8]	3.3 [1.4–8.0]
Chills	19 (2.3%) [1.5–3.6%]	9 (2.3%)	8 (5.9%)	2 (0.7%)	**0.004**	2.3 [0.7–8.2]	1.4 [0.9–2.2]	3.5 [1.0–12.0]
Secondary endpoints								
Urosepsis	5 (0.6%) [0.3–1.4%]	2 (0.5%)	3 (2.2%)	0 (0%)	**0.02**	∞ [0.1–∞]	4.4 [0.7–26]	∞ [0.6–∞]
Positive urine culture	21 (2.6%) [1.7–3.9%]	7 (1.8%)	11 (8.1%)	3 (1.0%)	** < 0.0001**	1.7 [0.4–6.5]	4.6 [1.8–11.6]	7.8 [2.2–27.4]
Positive blood culture	13 (1.6%) [0.9–2.7%]	5 (1.3%)	6 (4.4%)	2 (0.7%)	**0.02**	1.8 [0.4–9.3]	3.5 [1.1–11.3]	6.4 [1.3–31.1]

All *p* values written in bold demonstrate the the difference are statistically significant for purpose of better readability

Risk ratio estimates (RR) with 95% confidence intervals [CI]

*C-arm* ciprofloxacin arm, *F* Fosfomycin arm, *BF* bactrim plus fosfomycin arm, *UTI* urinary tract infection, *∞* infinite

### Microbiology

A urine culture was collected for all 37 patients who presented signs or symptoms of postinterventional infection. Of these, 21 samples tested positive. The most commonly isolated pathogen in the urine cultures was *E. coli* (n = 14, 67%). There were no significant differences in the isolated pathogens across the groups. No patient showed polymicrobial infection.

Blood cultures were positive in 13 samples, including nine patients with concomitant bacteruria (five *E. coli*, two *Klebsiella pneumoniae*, one *Klebsiella variicola*). One patient showed discrepant organisms in urine and blood samples. Among the positive samples, *E. coli* was the most common isolated microorganism (46%) followed by *Klebsiella* spp. (23%). The distribution of the isolated microorganisms was not significantly different across patient groups.

The incidence of microbiologically confirmed postinterventional UTIs was 2.6% (95% CI 1.7–3.9%), again with a significant difference between the groups C (1.8%), F (8.1%) and BF (1.0%) (p < 0.0001). Consistently, the incidence of bacteremia was 1.3%, 4.4% and 0.7% in the C, F and BF arm, respectively (p = 0.02).

### Factors associated with urinary tract infections

Increased BMI, intake of oral anticoagulation and presence of diabetes mellitus II increased the chance of having UTI in significant manner, while previous prostate biopsy seemed to have the opposite effect (Table 3).

**Table 3:** Multiplicative effects of potential risk factors on the odds of infection after prostate biopsy

Parameter	OR [95% CI]—ULR	p	OR [95% CI]—MLR	p
Age: for each year	0.98 [0.93, 1.02]	n.s	1.00 [0.95, 1.05]	n.s
BMI: for each kg/m^2^	1.09 [1.01, 1.19]	**0.04**	1.11 [1.02, 1.21]	**0.02**
PSA: for each µg/l LmL	1.00 [1.00, 1.00]	n.s	1.01 [0.97, 1.03]	n.s
Prostate volume MRI: for each cm^3^	1.00 [0.98, 1.01]	n.s	1.00 [0.98, 1.01]	n.s
Previous prostate biopsy: yes vs no	0.46 [0.19, 0.99]	**0.05**	0.37 [0.13, 0.89]	**0.02**
Oral anticoagulation: yes vs no	110.63 [8.79, 15,371.17]	** < 0.001**	223.10 [14.65, 33,525.30]	** < 0.001**
Diabetes mellitus II: yes vs no	21.49 [1.71, 269.56]	**0.02**	15.33 [0.97, 236.68]	**0.05**
Prostatitis: acute/mixed vs no	2.03 [0.53, 5.67]	n.s	2.62 [0.65, 8.16]	n.s
Cancer: yes vs no	0.84 [0.44, 1.63]	n.s	0.83 [0.38, 1.82]	n.s

All *p* values written in bold demonstrate the the difference are statistically significant for purpose of better readability

odds ratio estimates (OR) with 95% confidence intervals [CI] and p values from univariate (ULR) and multivariate (MLR) logistic regressions

The histological detection of a prostatitis was documented in 7.2% of all biopsies with an equal distribution of acute, chronic and mixed infections. Remarkably, prostatitis was not associated with an increased rate in consecutive postinterventional infection (Table 3).

## Discussion

TRUS-PB is the most frequent method for diagnosing prostate cancer nowadays and will likely continue to be in the foreseeable future [4, 5]. The main drawback of TRUS-PB is the high post-interventional infection rate, which was altogether 4.5% in our cohort and is comparable to the available literature. Some authors even report a post-intervention infection rate up to 7% [3, 17]. Ever changing bacterial resistance and the advent of MRI-TRUS fusion biopsy technique(s) may be contributing factors to this variability. Consequently, periinterventional antibiotic prophylaxis remains a crucial topic.

The EAU guidelines suggest several measures to reduce infections: rectal cleansing with povidone-iodine, rectal swab cultures for targeted antibiotic prophylaxis, the combination of antibiotics termed augmented prophylaxis as well as a transperineal approach [18].

In our study the combination of fosfomycin and TMP/SMX as augmented prophylaxis for 24 h was with 2.1% non-inferior vs 3.3% infection rate associated with ciprofloxacin prophylaxis for 72 h.

Reducing antibiotic exposure can prevent urinary tract infections, possibly by preserving the integrity of the intestinal flora [10]. Unfortunately, fosfomycin alone proved to be insufficient with 13.2%. Antibiotic resistance couldn’t fully account for the prophylaxis failures of the three regimens studied. Indeed, only 86%, 45%, and 67% of the breakthrough infections were resistant to the C, FMT, and TMP/SMX, respectively. Similar results were observed in the blood cultures. Therefore, pharmacokinetic and pharmacodynamics parameters appear to be equally important.

FMT seems to be ideal for prophylaxis due to the overall low resistance rates [19] and suitable prostate penetration [20], as demonstrated by reducing the risk of infectious complications following TRUS-PB compared with fluoroquinolones [12]*.* FMT has excellent activity against E. coli, including ESBL-producing and fluoroquinolone-resistant strains, and has a minimal impact on normal gastrointestinal flora. In 2021, 97.8% of *E. coli* were susceptible to fosfomycin at our institution, which compares to 87.1% and 76.7% susceptibility to ciprofloxacin and TMP/SMX, respectively. Yet, FMT alone exhibited the highest failure rate of all regimens tested in our patients. Taking FMT 2.5 h before the procedure might not be sufficiently early to ensure protective tissue concentrations at the start of the procedure. Some studies suggest optimal tissue concentrations require oral dosing 1–4 h prior to prostatic procedures [21, 22]. Thus, there seems to be potential for improvement and perhaps changing the time of administration to e.g. 3 or 4 h before procedure should be considered. Interestingly, data on proper timing and regimen are inconsistent in the literature ranging between 1 and 2 h and night before the procedure, often with second dosis applied mostly between 24 and 48 h after the procedure [15]. Noticeably, this concerns also other regimens as there is general lack of consensus regarding the duration of antibiotic prophylaxis preceding biopsy, with studies proposing durations ranging from 1 to 7 days [23].

Expanded antibiotic protocols can consist of either a broad-spectrum antibiotic or the use of multiple antibiotics, both being a selective force for emergence of multiresistant pathogens. The successful performance of the augmented prophylaxis in our study might be due to the combination of high susceptibility of urinary bacteria to FMT and rapid tissue penetration of TMP/SMX. However, even a 24-h prophylaxis couldn’t completely prevent infections with susceptible organisms. The presence of comorbidities such as diabetes mellitus, cardiac valve replacement, chronic obstructive pulmonary disease, immunosuppression, or benign prostatic hyperplasia have been variably reported to increase the risk of post TRUS-PB complications.

Notably in our population, higher BMI was significantly associated with infection, possibly due to lower tissue concentrations in overweight men. On the other hand, a history of prior prostate biopsies was correlated with lower infection rates in our study. This might result from a selection bias, as patients with past infectious complications undergo transperineal biopsies. This points to the existence of a subgroup of patients with an intrinsic higher complication rate. In line with earlier studies, anticoagulants and diabetes mellitus were also correlated with higher infection rates [24, 25].

The documentation of asymptomatic prostatitis in histological findings in 7.2% of all biopsies is notable and aligns with other reports [26]. Importantly, prostatitis at baseline did not increase the likelihood of a postinterventional infection, which suggests that different bacterial species might be causative.

The present study has several limitations. First and foremost the study is a retrospective analysis, thus results should be interpreted with caution. We can’t exclude potential biases such as unreported complications with underestimated infection rates, yet without falsifying the comparison between the three regimens. We also did not include a group with TMP/SMX prophylaxis alone due to high resistance rates in our population, so we cannot quantify the relative performance of the two compounds in the augmented prophylaxis. We did not monitor compliance with taking the peri-interventional prophylaxis, however this should also have negligible effect on the comparison between the groups.

The strengths of the study include the large sample size and the comprehensive data set including clinical and microbiological endpoints and resistance data. As almost all biopsies were performed by very experienced single physician, high quality and consistence were provided, as documented by the similar number of prostate biopsies throughout all groups. Consequently, we assert with reasonable assurance that the physician’s performance cannot be deemed a source of bias in our study.

## Conclusion

In conclusion, the change from FQ to FMT alone for peri-interventional antibiotic prophylaxis for transrectal prostate biopsies resulted in a significant increase in post-intervention infections. On the other hand, the combination of FMT and TMP/SMX was at least as protective as FQ without evident adverse events. These conclusions should be interpreted with caution given retrospective nature of our study and lacking comparison with TMP/SMX group alone.

## Data Availability

The data used in this study are available (upon irreversible anonymization and in accordance with swiss law regulations) on request from the corresponding author.
